# Three-Dimensional Path Planning Based on Six-Direction Search Scheme

**DOI:** 10.3390/s24041193

**Published:** 2024-02-12

**Authors:** Kene Li, Liuying Li, Chunyi Tang, Wanning Lu, Xiangsuo Fan

**Affiliations:** 1 School of Automation, Guangxi University of Science and Technology, Liuzhou 545006, China; likene@163.com (K.L.); lly_xxxx@163.com (L.L.); tangchunyi@gxust.edu.cn (C.T.); lwn01010@163.com (W.L.); 2 Guangxi Engineering Research Center for Mechanism and Control Technology of Mobile Robots, Liuzhou 545006, China

**Keywords:** 3D path planning, neural networks, collision energy, autonomous underwater vehicles

## Abstract

In order to solve the problem of how to perform path planning for AUVs with multiple obstacles in a 3D underwater environment, this paper proposes a six-direction search scheme based on neural networks. In known environments with stationary obstacles, the obstacle energy is constructed based on a neural network and the path energy is introduced to avoid a too-long path being generated. Based on the weighted total energy of obstacle energy and path energy, a six-direction search scheme is designed here for path planning. To improve the efficiency of the six-direction search algorithm, two optimization methods are employed to reduce the number of iterations and total path search time. The first method involves adjusting the search step length dynamically, which helps to decrease the number of iterations needed for path planning. The second method involves reducing the number of path nodes, which can not only decrease the search time but also avoid premature convergence. By implementing these optimization methods, the performance of the six-direction search algorithm is enhanced in favor of path planning with multiple underwater obstacles reasonably. The simulation results validate the effectiveness and efficiency of the six-direction search scheme.

## 1. Introduction

In recent years, due to the abundance of marine resources, robotics scholars have been paying more and more attention to underwater exploration. Autonomous underwater robots (AUVs) have been widely used because they can combine advanced intelligent algorithms for unmanned operation, and their related technologies have been rapidly developed. Due to the complex underwater environment with various types of obstacles and random locations, underwater path planning in an underwater multi-obstacle environment has become a necessary technology for an AUV to accomplish the related tasks safely [1,2]. 

Underwater 3D path planning to find a path without collision from the start point to the goal point is a vital and challenging issues because there are an infinite number of paths which are subject to physical constraints in a 3D space. Path planning can generally be divided into global path planning and local path planning according to the known information about the environment [3]. For global path planning, all the information about the environment is known, which can be used to complete the planning. Many algorithms are proposed for global path planning, such as the A* algorithm [4,5], Dijkstra algorithm [6], Grid-based methods and so on. While the information for local path planning is not totally known, some environmental information must be collected in real time by sensors to determine the location of local obstacles and then to carry out path planning. Some typical algorithms are the Dynamic window approach (DWA) [7], Artificial potential field method (APF) [8,9], Rapidly exploring random tree algorithm (RRT) [10,11] and so on. 

Most of the algorithms proposed above are suitable for 2D environments. In recent years, scholars have found that there are related intelligent algorithms available for 3D path planning, such as bio-heuristic intelligence optimization algorithms, neural networks and other algorithms [12]. Bio-heuristic intelligent optimization algorithms mainly include particle swarm optimization (PSO) [13,14], ant colony optimization (ACO) [15,16], genetic algorithm (GA) [17,18] and so on. 

Phung et al. [13] adopted the PSO to directly implement the constraints related to turning angle and climb angle through the elevation and azimuth angles of the spherical vectors. However, some premature convergence phenomena occurred. Shao et al. [14] exploited the PSO with chaotic logistic mapping to promote the initial distribution of particles, as well as the optimality of the solution. Chen et al. [15] combined the ACO algorithm and APF algorithm to improve convergence and eliminate local minimum problems. Yan et al. [16] improved the transfer probability and pheromone update strategy to solve the problem of local optimal solution and the slow convergence speed of the traditional ant colony algorithm. Hao et al. [17] proposed an adaptive genetic algorithm to prevent path individuals from falling into the deadlock state during the generation process and reduce the time of global path generation. Tao et al. [18] designed reasonable crossover and mutation adaptive probability models to converge quickly. The bio-heuristic intelligent optimization algorithms usually display higher efficiency. But there are also problems such as high computational complexity and inefficient utilization of target information. 

Neural networks have been developed rapidly and have presented many new-type models in recent years [19,20]. They are capable of performing complex computational tasks and can also be applied to path planning. Wang et al. [21] submitted a sampling-based path planning framework that utilized a deep neural network to predict feasible paths and output promising regions. Guo et al. [22] adopted the Li activation function and by computing the time-varying pseudoinverse of the Jacobian matrix, the resultant ZNN model was applied to redundant manipulator kinematic control. Kroumov et al. [23] described obstacles using energy functions defined by neural networks and different path generating equations were used, depending on the path points inside or outside the obstacles. Alex et al. [24] applied a neural network to determine the orientation of an object for a robotic arm’s grasping task. 

Based on the above analysis, bio-intelligent algorithms in 3D environments have more efficient computing efficiency but require more executing time. Meanwhile, neural networks are also widely used in 3D path planning. Li et al. [25] proposed a four-direction algorithm that designed a simpler search method and required fewer iterations. Therefore, inspired by the four-direction algorithm, this paper presents a six-direction search scheme for path planning in 3D environments. Specifically, the scheme solves the problem of path planning for AUVs with multiple obstacles underwater by reducing the iteration step length and reducing the path nodes. 

This paper is organized as follows. Section 2 presents the neural network-based six-direction path planning scheme. The simulations of various situations are given in Section 3. The proposed algorithm performs physical verification on the robotic arm in Section 4. Finally, the conclusion is given in Section 5.

## 2. Problem Description

Finding a collision-free path in 3D space became a challenging issue with the increase in search space and number of objects [26]. This paper presents a path planning algorithm based on a neural network and discusses its efficiency and realizability. The presented algorithm can be designed as follows. Firstly, the obstacle can be described as a polyhedron or a ball. Secondly, the collision energy of the obstacle is obtained based on the neural network. Thirdly, to avoid generating a too long path, the algorithm introduces path energy, i.e., the sum of squared distances between adjacent nodes in the path. Finally, the collision energy and the path energy are weighted and summed, and the generated path with the smallest sum is selected as the final path.

### 2.1. Formulation of Collision Energy Function

**Figure 1:**
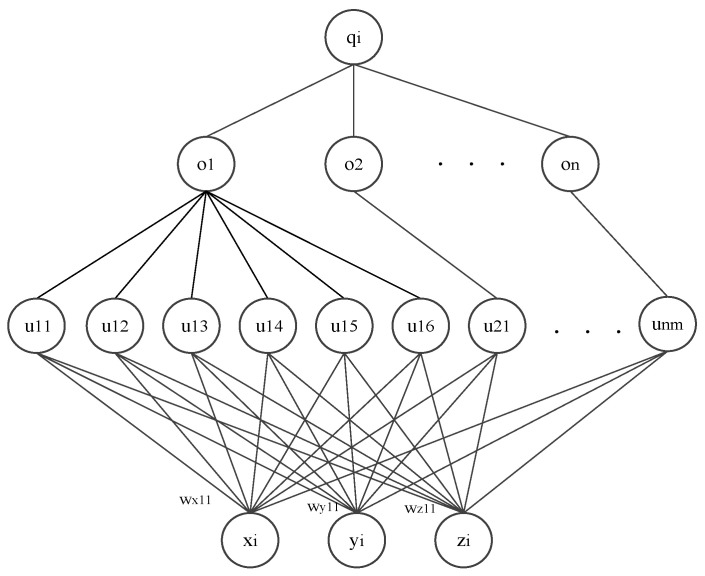
The neural network structure for the obstacles’ energy.

Generally, in a known 3D environment, the information of the position and shape of an obstacle can be used, e.g., a stationary obstacle can be described as a polyhedron or a ball. In order to formulate the distances between paths and obstacles, the distances are modeled as collision energy based on the neural network approach. Thus, the energy distribution related to obstacles in the environment is obtained, as shown in Figure 1. The neural network consists of an input layer, a hidden layer and an output layer.

In the input layer, the three neurons *x_i_*, *y_i_*, *z_i_* demonstrate the coordinates of a point in 3D space. The neurons of the first hidden layer depict the spatial constraints of the obstacles, and those of the second hidden layer function describe the collision energy output of an obstacle. In the output layer, the neuron represents the total collision energy of a 3D space point, i.e., the sum of collision energy of all obstacles to this point.

Specifically, the first hidden layer is determined by obstacles in the environment, and the corresponding polyhedron of an obstacle is constrained by a set of sides, and thus can be described as

(1) $$w_{x}x+w_{y}y+w_{z}z+\sigma =0$$

where w_x_, w_y_, w_z_, σ are the side function coefficients. 

Therefore, if an obstacle is a cuboid, its six-side inequality is expressed as follows:

(2) $$\left\{\begin{array}{c} w_{xj1}x_{i}+w_{yj1}y_{i}+w_{zj1}z_{i}+\sigma_{uj1}\geq 0\\ w_{xj2}x_{i}+w_{yj2}y_{i}+w_{zj2}z_{i}+\sigma_{uj2}\geq 0\\ w_{xj3}x_{i}+w_{yj3}y_{i}+w_{zj3}z_{i}+\sigma_{uj3}\geq 0\\ w_{xj4}x_{i}+w_{yj4}y_{i}+w_{zj4}z_{i}+\sigma_{uj4}\geq 0\\ w_{xj5}x_{i}+w_{yj5}y_{i}+w_{zj5}z_{i}+\sigma_{uj5}\geq 0\\ w_{xj6}x_{i}+w_{yj6}y_{i}+w_{zj6}z_{i}+\sigma_{uj6}\geq 0 \end{array}\right.$$

The coefficients *w_xjk_*, *w_yjk_*, *w_zjk_* are taken as weight values of the input neurons *x_i_*, *y_i_*, *z_i_* to the *jk*-th hidden layer neuron with *k =* 1, 2, 3, 4, 5, 6 and *σ_ujk_* as its threshold. 

**Figure 2:**
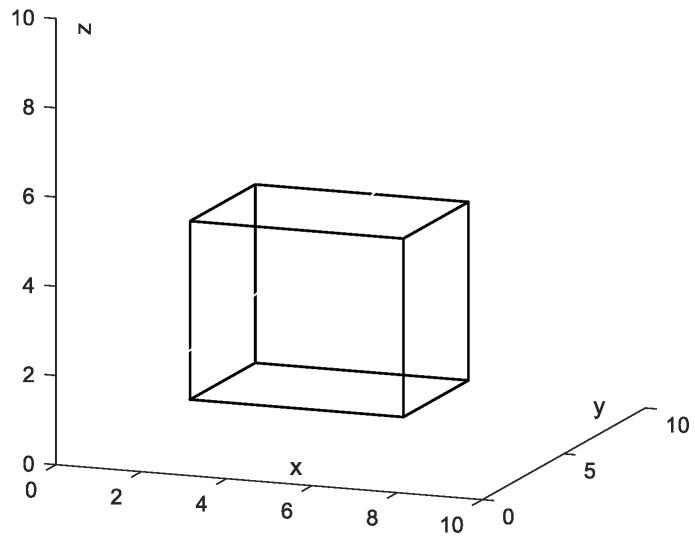
A cuboid obstacle.

There is a cuboid obstacle in space as shown in Figure 2, where the thresholds of *w_x_*, *w_y_*, *w_z_*, *σ* are as follows:

$$\begin{array}{c} w_{x1}=1,\sigma_{j1}=-2,w_{y1}=w_{z1}=0;\\ w_{x2}=-1,\sigma_{j2}=8,w_{y2}=w_{z2}=0;\\ w_{y3}=1,\sigma_{j3}=-3,w_{x3}=w_{z3}=0;\\ w_{y4}=-1,\sigma_{j4}=7,w_{x4}=w_{z4}=0;\\ w_{z5}=1,\sigma_{j5}=-1,w_{x5}=w_{y5}=0;\\ w_{z6}=-1,\sigma_{j6}=5,w_{x6}=w_{y6}=0; \end{array}$$

The first hidden layer neuron function for the *k*-th side of *j*-th obstacle is taken as

(3) $$u_{jk}(x_{i},y_{i},z_{i})=\frac{1}{1+e^{-(w_{xjk}x_{i}+w_{yjk}y_{i}+w_{zjk}z_{i}+\sigma_{ujk})/T}}$$

where *T* is a positive design parameter. In addition, if the *j*-th obstacle is a spherical obstacle, the first hidden layer neuron function is

(4) $$u_{j}(x_{i},y_{i},z_{i})=r^{3}-{\left(x_{i}-C_{jx}\right)}^{2}-{\left(y_{i}-C_{jy}\right)}^{2}-{\left(z_{i}-C_{jz}\right)}^{2}$$

where *r* is the radius, and (*C_jx_, C_jy_, C_jz_*) are the center coordinates of the *j*-th spherical obstacle.

The second hidden layer neuron function for the *j*-th obstacle with *k* sides is taken as

(5) $$o_{j}(u_{j1},u_{j2},\cdots ,u_{jk})=\frac{1}{1+e^{-(\sum_{i=1}^{k}u_{ji}-\sigma_{oj})/T}}$$

where *σ_oj_*= *k* − 0.5 (specifically, for the spherical obstacle *σ_oj_* = 0.5).

For a 3D space with *n* obstacles, the energy of each point can be obtained, and the output layer function is

(6) $$q_{i}(o_{1},o_{2},\cdots ,o_{n})=\sum_{j=1}^{n}\left(o_{1},o_{2},\cdots ,o_{n}\right)$$

### 2.2. Formulation of Path Energy Function

**Figure 3:**
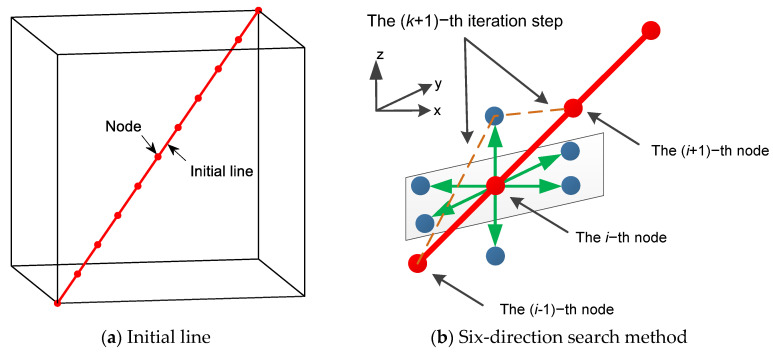
Introduction to the six-direction search method. The green arrows indicate the direction the mobile agent. The blue balls indicate the six directions in space.

We take the straight line between the start point and the goal point as the initial path. The initial path is divided into *s* line segments of the same length, and then there are *s* + 1 nodes in the path, as shown in Figure 3a. During the search period, the path nodes would be driven away from the high collision energy point to obtain a collision-free path. However, a longer path may be generated for lower collision energy in order to avoid obstacles. But too long of a path would not be optimal for path planning. For evaluation convenience, the sum of squares of two adjacent nodes in the path is taken as the path energy. In order to prevent the generated path from being too long, in this paper, we take the path energy along with the obstacle energy into consideration.

Based on the above discussion, the path energy can be obtained:

(7) $$L=\sum_{i=0}^{s-1}\left[{\left(x_{i+1}-x_{i}\right)}^{2}+{\left(y_{i+1}-y_{i}\right)}^{2}+{\left(z_{i+1}-z_{i}\right)}^{2}\right]$$

where *L* denotes the total path energy, *s* denotes the total number of the line segments divided from the initial path, *x_i_, y_i_, z_i_* denote the coordinates of the *i*-th path node and (*x*_0_, *y*_0_, *z*_0_) denote the coordinates of the starting path node.

Different from other search algorithms, a six-direction search (SDS) algorithm is proposed in 3D space to lessen the path search computation, as shown in Figure 3b. By setting the search step length δ, for the *i*-th node, the SDS can be obtained as follows:

(8) $$\begin{array}{l} x_{i}{\;}^{\left(k+1\right)}=x_{i}{\;}^{\left(k\right)}+\delta \cdot \cos(j\cdot \pi /2)\cdot \sin(k\cdot \pi /2)\\ y_{i}{\;}^{\left(k+1\right)}=y_{i}{\;}^{\left(k\right)}+\delta \cdot \sin(j\cdot \pi /2)\cdot \sin(k\cdot \pi /2)\\ z_{i}{\;}^{\left(k+1\right)}=z_{i}{\;}^{\left(k\right)}+\delta \cdot \cos(k\cdot \pi /2) \end{array}$$

where the superscript (*k* + 1)-th denotes the (*k* − 1)-th iteration and *j* = 1, 2, 3, 4, *k* = 2,3,4. The newly generated six nodes are linked with the (*i* − 1)-th and (*i* + 1)-th nodes, respectively, to form new paths. The obstacle energy and path energy of the new paths are calculated. Together with the results of the *k*-th path, the path with the minimum energy is selected as the optimal path.

Furthermore, in order to improve the problem of a large number of iterations and premature convergence of the total energy caused by the fixed step length, we employ variable step length. The variable step length approach is to search quickly by using larger step lengths in the pre-search phase and smaller step lengths are employed with the mobile agent being far away from the obstacle in the post-search phase. The variable step length $\delta_{p}$ is presented as follows:

(9) $$\delta_{p}=\left|\right|Q|{|}_{\infty }\cdot \delta$$

where $\;Q=[q_{1},q_{2},\cdots ,q_{n}]$ for a path with *n* nodes, the symbol $||\cdot |{|}_{\infty }$ denotes the infinity norm of a vector and δ is an initial fixed step length. 

This article uses a neural network structure to model the obstacle energy and then calculates the energy distribution model of obstacles in the 3D space. A higher obstacle energy indicates the presence of obstacles or proximity to obstacles, while a lower obstacle energy indicates a greater distance from obstacles. The introduction of path energy can avoid the generated path being too long. By using obstacle energy and path energy, a collision-free and low-energy path can be obtained.

## 3. Simulation Studies

In this section, path planning is performed in a given 3D environment using the proposed SDS method. In the presence of a cuboid obstacle in space, the performance of the algorithm with different iteration step lengths is analyzed and discussed. Then, the results are applied to a multi-obstacle situation. The MATLAB simulation is conducted in a 3D modeling environment.

### 3.1. Fixed-Iteration Step Length Studies

**Figure 4:**
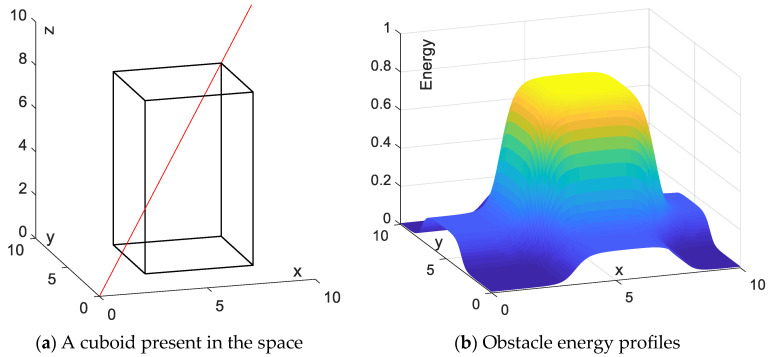
Modeling of individual obstacle energy in space. The red line is a straight line from start to end.

For discussion convenience, a cuboid obstacle is introduced and the fixed iteration step length is used for the simulation conducted in a 3D space with X ∈ [0, 10], Y ∈ [0, 10], Z ∈ [0, 10]. The cuboid obstacle vertex is set as {(3,3,0), (3,8,0), (8,8,0), (8,3,0), (3,3,8), (3,8,8), (8,8,8), (8,3,8)}. The red line is a straight line from start to end. The mobile agent is assumed to move from the start point (0,0,0) to the goal point (10,10,10), as shown in Figure 4a. Figure 4b illustrates the collision energy distribution of the cuboid obstacle.

**Figure 5:**
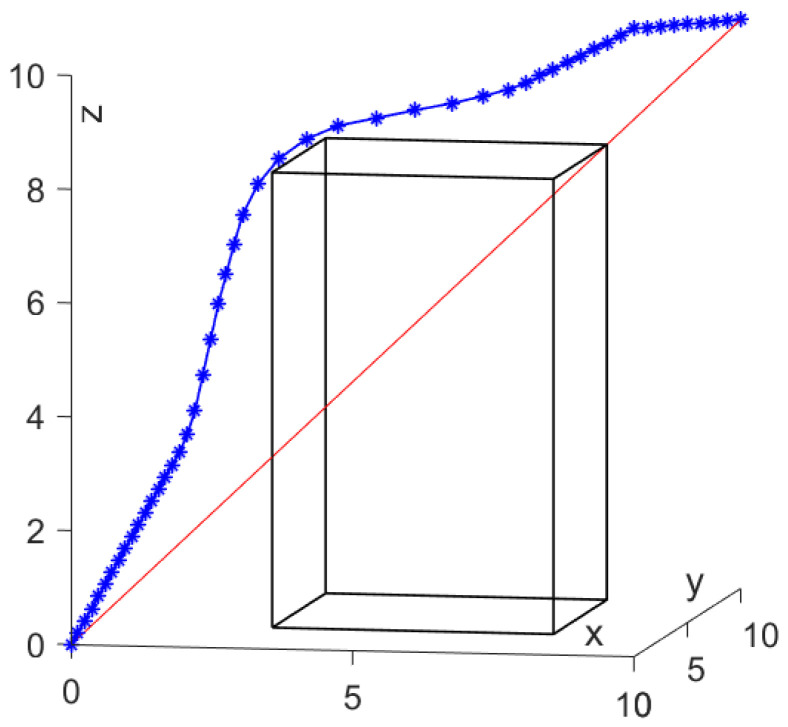
Path planning by SDS scheme with fixed δ = 0.1.

For the first case, the iteration step length δ = 0.1 is used to test the effectiveness of the scheme. In Figure 5, the red line represents the shortest distance from the start point to the goal point, and the blue line represents the actual search path using the SDS. It is found that the proposed SDS scheme allows the mobile agent to move from the initial position to the target position, and the generated path can avoid obstacles without collision.

**Figure 6:**
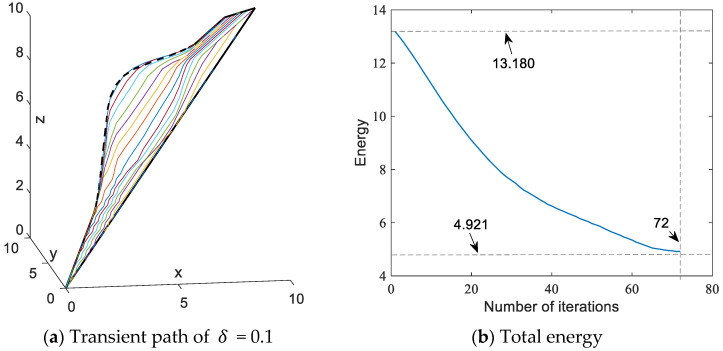
Paths synthesized by SDS with δ = 0.1. The colored lines indicate the generated transient paths during the move.

The transient paths and total energy variations for the corresponding paths are shown in Figure 6. As seen in Figure 6b, the total energy (i.e., the weighted sum of the collision energy of the obstacle and the distance energy between adjacent points) decreases from the initial state of 13.180 to the final state 4.921, and the number of iterations is 72. The executing time for this simulation is 0.049669. The simulation results demonstrate that the SDS algorithm works properly to avoid a stationary obstacle. It is worth pointing out that the SDS algorithm converges prematurely in the case of a larger step length δ = 0.1, resulting in the final path being close to the rectangular obstacle.

**Figure 7:**
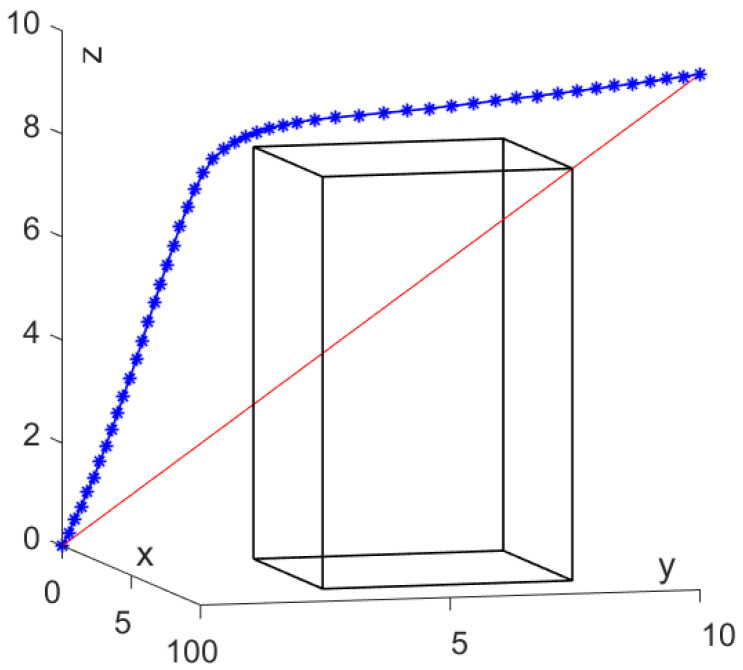
Path planning by SDS scheme with fixed *δ* = 0.01.

To improve this phenomenon of early convergence, a smaller iteration step length δ = 0.01 is employed to further validate the SDS scheme, and the result is shown in Figure 7. As seen from Figure 7, the generated path can reach the target point without collision and the critical point does not closely approach the cuboid obstacle, which makes the path more feasible in practice. 

**Figure 8:**
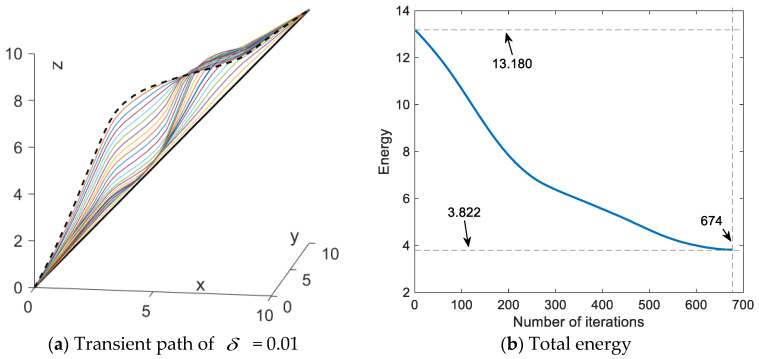
Paths synthesized by SDS with *δ* = 0.01.

Figure 8 illustrates the transient paths and their total energy variations. In case *δ* = 0.01, the total energy decreases from an initial state of 13.180 to a final state of 3.822 with 674 iterations. Compared to the *δ* = 0.01 case, with *δ* = 0.01 employed, the number of iterations increased by 836.1% but the total energy decreased by 22.3%, and the scheme generated a smoother path. These results can confirm that premature convergence can be improved by reducing the iteration step length. However, it is worth noting that reducing the iteration step length will greatly increase the number of iterations and the amount of computation will also increase a lot. 

### 3.2. Variable Iteration Step Length Studies

**Figure 9:**
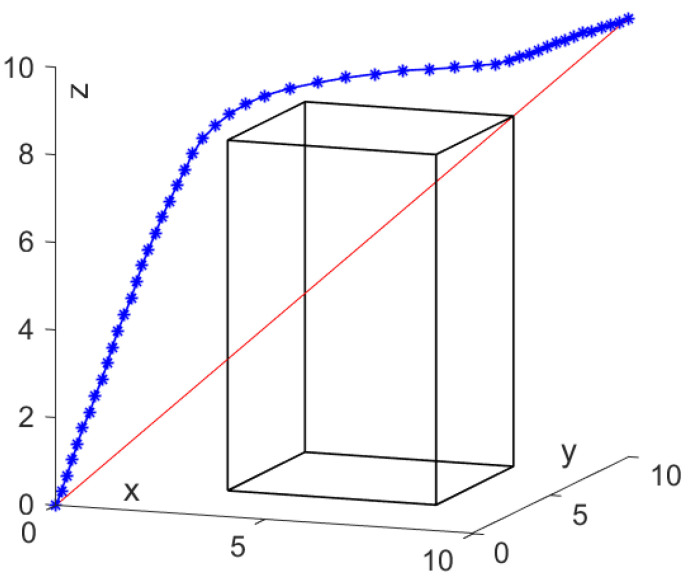
Path planning by SDS scheme with the variable step length.

There are fewer iterations with iteration step length δ = 0.1 and better obstacle avoidance performance with step length δ = 0.01. In order to obtain the advantages in both cases, we further designed the step length. Through the above discussion and analysis, a variable step length is presented that depends on the collision energy based on the advantages of the different step lengths. The variable step length is designed as $\delta_{p}=|\left|Q\right|{|}_{\infty }\cdot \delta_{0}$, where *Q* denotes the energy vector of the obstacle at the nodes and $\delta_{0}$ denotes the initial step length and is related to the size of the obstacle. For testing and comparison purposes, $\delta_{0}$ is set to 0.1. Figure 9 depicts the final path for the variable step length scheme, where the critical point is not close to the rectangular obstacle, similar to the case of δ = 0.01, but with fewer iterations than the case of δ = 0.01.

**Figure 10:**
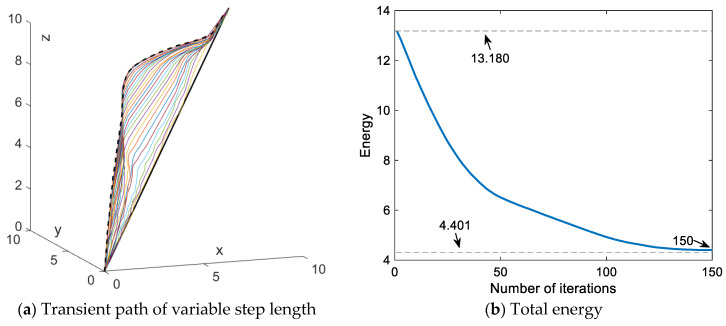
Paths synthesized by SDS with variable step length.

Figure 10 describes the transient profiles and total energy variations for the corresponding paths. The collision energy decreases as the number of iterations increases and the variable step length $\delta_{p}$ also becomes smaller as the collision energy decreases. As can be seen from Figure 10b, the total energy decreases from the initial state 13.180 to the final state 4.401 with 150 iterations. Compared to the fixed δ = 0.1, the scheme increases the number of iterations by 108.3%. Additionally, it decreases the total energy of the final convergence point by 10.6%. On the other hand, compared to the fixed δ = 0.01, it decreases the number of iterations by 77.7% and increases the total energy of the final convergence point by 15.1%. This situation implies that employing variable step length significantly diminishes the number of iterations and can improve premature convergence. However, the results of this approach are not satisfactory.

It is worth noting that the iteration numbers of the scheme with different step lengths substantially vary when the total energy decreases to about half from 13.180, which is shown in Table 1. Specifically, it takes 35 iterations to decrease to 7.055 for the fixed step length δ = 0.1, 244 iterations to 7.013 for the fixed step length δ = 0.01 and 41 iterations to 7.036 for the variable step length. 

### 3.3. Path Node Reduction Studies

By varying the step length or adjusting the step length in the experiment, better experimental results can be achieved. This approach reduces the number of iterations, resulting in an improved search performance. Considering the larger dimensionality of the 3D space, higher number of SDS nodes and higher algorithm complexity, a method for a reduction in path nodes is presented to optimize this problem. The approach takes into account the specific challenges posed by these factors and provides a more efficient solution.

Through reducing the path nodes, the scheme can efficiently and rapidly search the collision-free path. And it can effectively reduce the number of iterations required as the path nodes decrease. To optimize the path further, adding more nodes to the path is considered. For comparison purposes, the number of nodes can be supplemented to 100, as in other cases. For the path continuity, the nodes are supplemented to the path by linear interpolation. The pseudo-code for the Algorithm 1 is as follows:
**Algorithm 1** Point Reduction Method1 For all i loops 2  If i is even number 3   x1(i) = (x(i/2) + x((i/2) + 1))/2 4   y1(i) = (y(i/2) + y((i/2) + 1))/2 5   z1(i) = (z(i/2) + z((i/2) + 1))/2 6  else 7   x1(i) = x((i + 1)/2) 8   y1(i) = y((i + 1)/2) 9   z1(i) = z((i + 1)/2) 

**Figure 11:**
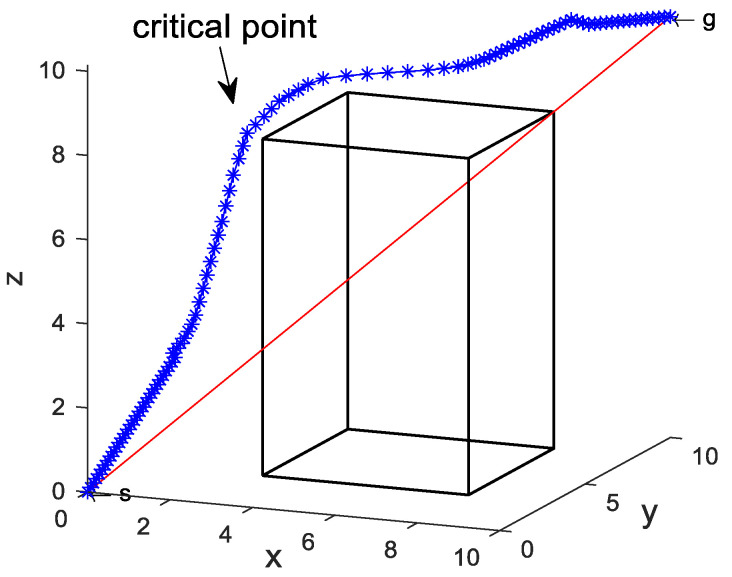
Path point reduction.

In Figure 11, the final path generated by the SDS algorithm of the reduced path nodes scheme is shown, where the critical point is not in close proximity to the obstacle.

**Figure 12:**
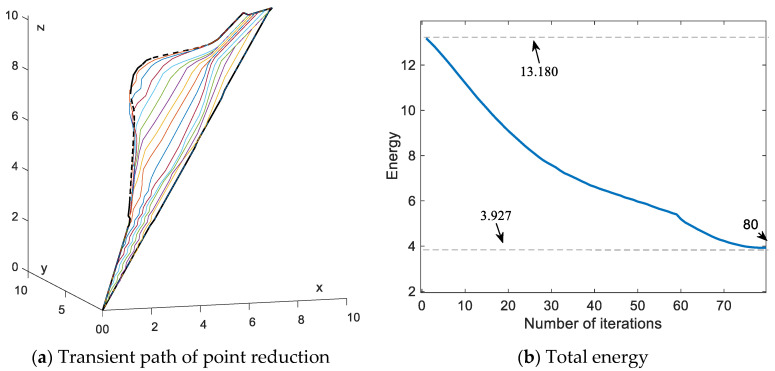
Paths synthesized by SDS with reduce path node.

In Figure 12, the transient profiles and variations in total energy are presented for the corresponding paths. It can be seen that the total energy decreases from the initial state of 13.180 to 3.927, which is closer to the total energy of 3.822 in the δ = 0.01 case. Compared to the variable step length scheme, this scheme reduces the number of iterations by 46.7% and the total energy of the final convergence point by 10.8%. The data reveal that the reduced path node scheme requires a lower number of iterations compared to the previous schemes. Additionally, it demonstrates a significant decrease in total energy when compared to Figure 6b and Figure 10b, and it can effectively improve the premature convergence.

With the comparison of the reduced path node scheme with the variable step length scheme, a significant improvement in the final convergence energy and a reduction in the number of iterations can be seen. Reducing the path node energy eventually converges to 3.927, which is smaller than 4.401 of the variable step length schemes. In addition, the number of iterations of the reduced path node scheme is 80, which is close to one-half the number of iterations for the variable step length. This implies that convergence of collision energy can be optimized by reducing the number of path nodes. Moreover, this scheme can further decrease the number of iterations. These results validate that reducing the path nodes serves as an effective and feasible alternative to the proposed SDS method.

The simulation data gained with different step lengths and methods are summarized in Table 2. After comparing them, they show that the path nodes reduction scheme is the best one.

### 3.4. Path Planning Studies for Multiple Obstacles

**Figure 13:**
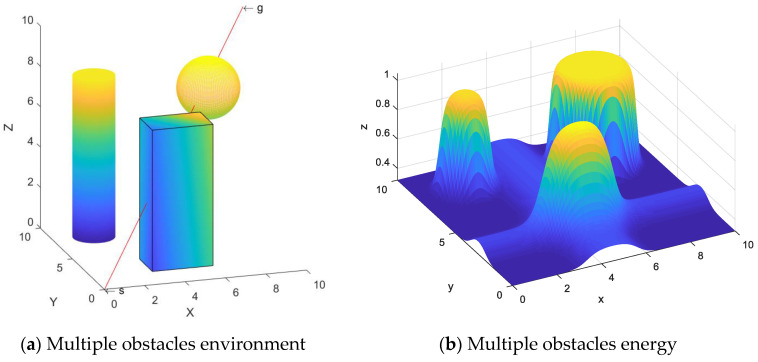
Energy modeling of multiple obstacles in space.

In this section, to further validate the effectiveness of the SDS scheme, multiple obstacles are considered in the 3D environment. Three obstacles are set typically as a cuboid, a cylinder and a sphere. In detail, the vertex, cylindrical base center or sphere-center coordinates of the obstacles are {(3,2,0), (6,2,0), (3,4,0), (6,4,0), (3,2,7), (6,2,7), (3,4,7), (6,4,7)} for the cuboid obstacle, {(2,8,0), *r* = 1, *h* = 8} for the cylindrical obstacle and {(7,7,7), *r* = 1.5} for the sphere obstacle, respectively. It is assumed that the mobile agent starts moving from the start point (0,0,0) and stops at the target point (10,10,10), as shown in Figure 13a. Figure 13b shows the energy of the obstacles.

**Figure 14:**
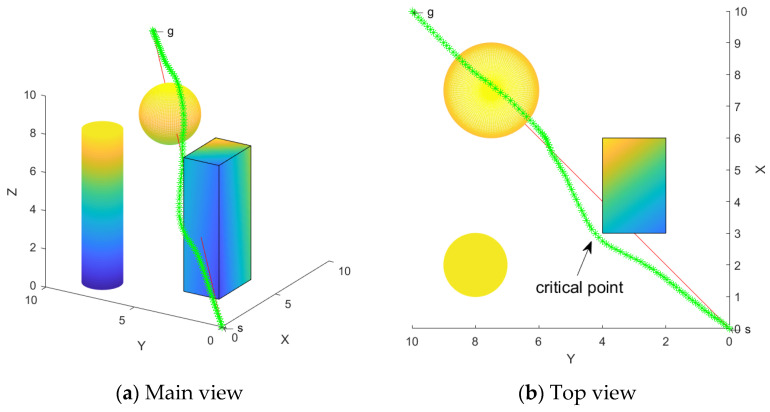
Multiple obstacle avoidance path.

The green line is the path generated using the SDS algorithm, and the simulation results are shown with two observation views, as seen in Figure 14. The simulation results demonstrate that the generated path is smooth and successfully avoids collision with obstacles. Moreover, the critical point remains at a certain distance from the obstacles, indicating the effectiveness of this scheme for the avoidance of multiple obstacles.

**Figure 15:**
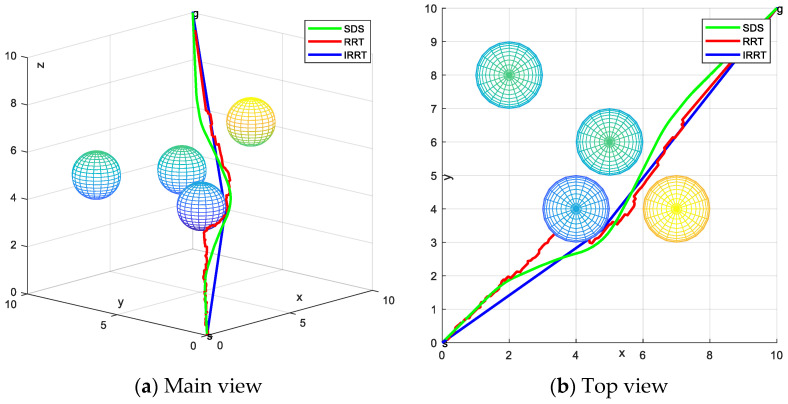
Paths generated by the different algorithms.

Remarks. For the purpose of this paper, to verify the feasibility of the proposed search scheme, the SDS algorithm is analyzed in comparison with the traditional RRT algorithm and the IRRT algorithm that was proposed in the literature [11]. Both the RRT algorithm and the SDS algorithm are comparable as they obtain new nodes by utilizing a node expansion strategy and find the final path after several explorations and expansions. Figure 15 shows the optimal paths obtained by the SDS, RRT and IRRT algorithms in the same case, with the start and goal points represented by the green and blue balls, respectively. As can be seen from the figure, in the same situation, the paths generated by all schemes can avoid obstacles to reach the target point. Compared to the paths generated by the SDS and IRRT, the RTT path is smoother. The proximity between the paths generated by RTT and IRRT and the obstacles creates a risk of collision. But the path generated by SDS is a certain distance from the obstacles, which does not generate a risk of collision and it is more in line with the requirements of the actual situation. 

The experimental data of the SDS, RRT and IRRT algorithms are presented in Table 3, where the number of iterations and running time of the three schemes are compared. The SDS algorithm is better than the RRT algorithm and IRRT algorithm in terms of the number of iterations and running time.

## 4. Physical Experiment Verification

**Figure 16:**
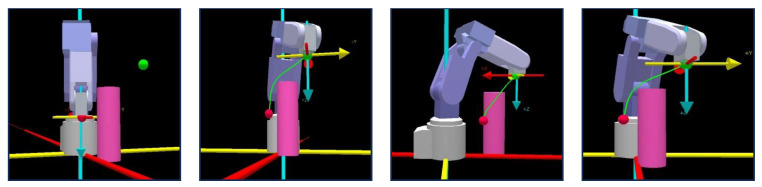
Simulation experiment.

The physical experiment verification includes two parts: physical robot model simulation and physical robot verification. The experiment is conducted on a Mitsubishi six-degree-of-freedom robotic arm RV-2F-D. The unit of each parameter in the experiment is in millimeters (mm). The starting coordinates of the robot end-effector (i.e., the red ball location) are (150,−50,170), and the target coordinates (i.e., the green ball location) are (350,150,370). For illustration convenience, only a cylindrical obstacle {(250,50,0), *r* = 20, *h* = 290} is set in the experimental space. To verify the path generated by the SDS method based on the simulative robotic arm RV-2F-D model, the final path nodes optimized by the SDS algorithm are input into the Mitsubishi robotic arm simulation software GX simulator. Then, the robotic arm performs path following, and the actual path is shown in Figure 16. From Figure 16, it can be seen that the robotic arm can follow a smooth path with obstacle avoidance. These simulation results verify that the scheme of the SDS algorithm is effective and feasible. 

**Figure 17:**
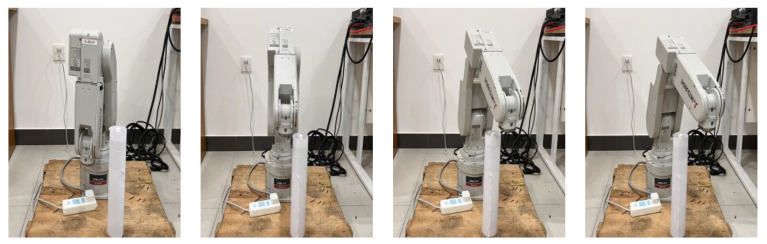
Main view of the robotic arm performing the path following.

**Figure 18:**
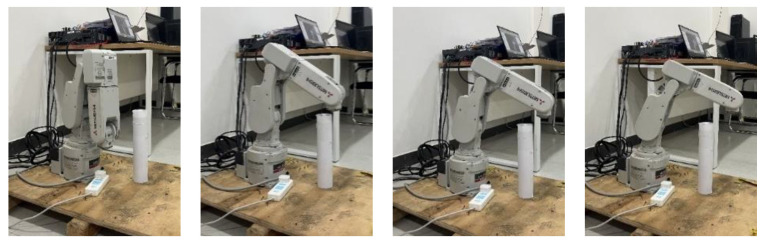
Side view of the robotic arm performing the path following.

To further validate the feasibility of the SDS algorithm, a physical experiment is conducted on the robotic arm RV-2F-D. Figure 17 and Figure 18 illustrate the transient profiles of the robotic motion from the front and side views, and it can be seen that the robotic arm successfully performs the path-tracking task without any lag. These results demonstrate that the path can be followed smoothly by the robotic arm and is suitable for physical applications. 

## 5. Conclusions

In a 3D underwater environment, an SDS strategy based on neural networks is proposed in this paper in order to make the paths planned by AUVs safer and more efficient when facing multiple obstacles. Neural networks are employed to construct collision energy models for stationary obstacles. The generated paths are optimized by adjusting the search step length and reducing the number of path nodes, which effectively decreases the number of iterations and total energy. Based on the simulation and physical verification, the results indicate that the proposed SDS algorithm successfully avoids obstacles and achieves efficient 3D path planning. For future research, potential improvements will include simplifying the path search direction, optimizing the iteration process, and extending the algorithm to underwater environments with dynamic obstacles. 

## Figures and Tables

**Table 1:** Iterations when the energy of different schemes falls to half of the total energy.

Improvement Measures	Energy Iteration Approximation	Iterations
Fixed step δ = 0.1	7.055	35
Fixed step δ = 0.01	7.013	244
Variable step length	7.036	41

**Table 2:** Simulation data.

Improvement Measures	Whether to Avoid Obstacles	Iterations	Total Energy	Computational Time	Whether or Not to Converge Prematurely
Fixed step δ = 0.1	Y	72	4.921	0.049669	Y
Fixed step δ = 0.01	Y	674	3.822	0.197290	N
Variable step length	Y	150	4.401	0.064872	Y
Path nodes reduction	Y	80	3.927	0.064124	N

**Table 3:** Experimental data.

Algorithms	Iterations	Computational Time
SDS	205	0.112469
RRT	359	4.301346
IRRT	329	0.469415

## Data Availability

No new data were created or analyzed in this study.
